# The Modified Yale Food Addiction Scale May Be Simplified and Diagnostically Improved: The Same Prevalence but Different Severity and Risk Factors of Food Addiction among Female and Male Students

**DOI:** 10.3390/nu14194041

**Published:** 2022-09-28

**Authors:** Edyta Charzyńska, Anna Brytek-Matera, Paweł A. Atroszko

**Affiliations:** 1 Faculty of Social Sciences, University of Silesia in Katowice, Bankowa 12, 40-007 Katowice, Poland; 2 Eating Behavior Laboratory (EAT Lab), Institute of Psychology, University of Wrocław, 50-527 Wrocław, Poland; 3 Institute of Psychology, University of Gdańsk, Bażyńskiego 4, 80-309 Gdańsk, Poland

**Keywords:** food addiction, the modified Yale Food Addiction Scale, addictions, validity, measurement invariance, prevalence, gender differences, personality, well-being

## Abstract

The Yale Food Addiction Scale (YFAS) is the most commonly used scale for measuring food addiction (FA). The previous approach to the YFAS and its subsequent versions assumed dichotomization of items, separating addiction symptoms and clinical significance items, and factorial validity testing on a subset of items. In this paper, we discuss the drawbacks associated with these procedures. In addition, we present a different analytical approach to investigate the validity of the modified YFAS (mYFAS) along with an alternative scoring method that overcomes limitations related to the previous approach. After establishing the structure of the mYFAS, we investigated the potential antecedents and consequences of FA separately for men and women. The sample consisted of 1182 Polish undergraduate students (613 women, 559 men, 10 missing values on gender) with a mean age of 20.33 years (*SD* = 1.68; range: 18–36). They were asked to complete self-report questionnaires measuring FA, personality traits (Big Five), self-esteem, narcissism, self-efficacy, social anxiety, loneliness, and well-being indicators. Due to the low content, factorial, and clinical validity, the first three items were excluded from the Polish version of the mYFAS. The six-item mYFAS demonstrated measurement invariance, allowing for meaningful comparisons between genders and yielded almost identical prevalence rates for men and women. The hierarchical multiple regression analysis showed that, narcissism, and social anxiety predicted FA in both genders, whereas important gender differences in antecedents were also noted. In addition, FA was associated with body mass index (BMI) and most of the well-being indicators, even after controlling for relevant variables. The findings suggest that our modified analytical approach allows researchers to measure FA using a valid, useful, and simple tool.

## 1. Introduction

### 1.1. Definition of Food Addiction

Despite a lack of conceptual clarity on food addiction (FA) and an explicit and agreed definition of this concept [1], FA is generally referred to as excessive consumption of highly processed foods (hyper-palatable food) that contain high combinations of sugar, salt, and fat [2,3,4]. Evidence suggests that highly palatable foods affect the brain and activate the same brain regions and the same neural circuits, just as with drugs of abuse [5,6]. Excessive food consumption might activate neural adaptation in reward circuitry, similar to drug dependency [7]. Thus, excessive exposure to highly palatable foods can induce behavioral responses that mimic those seen in drug-related addiction responses. According to the FA hypothesis, a person can be addicted to certain foods (rich in sugar, salt, and fat) and display addiction-like symptoms in response to some food [8]. Highly processed foods (refined grains, added sugars, sweeteners, fats, and salt) that share characteristics of drugs of abuse (e.g., high dose, rapid rate of absorption) are most associated with FA [4].

This abnormal pattern of excessive consumption is also defined as hedonic eating behavior involving consuming highly palatable foods in quantities beyond homeostatic energy requirements [9] to obtain pleasure or arousal or alleviate emotional or physical discomfort [10]. Some findings suggest that individuals with FA may turn to addictive food consumption as a coping strategy for increased negative emotions [11]. There also is support showing that many people self-medicate with comfort food [10] to improve mood and/or because of hypothetical reward deficiency as a result of a decrease in basal dopamine levels due to elevated substance use or food consumption [6].

The concept of FA remains controversial. Some researchers question whether food or eating can be addictive if it is necessary to human survival and whether there are particular foods that are “addictive” and, therefore, akin to drugs of abuse [12], while others point out the common biological (e.g., brain reward pathways), behavioral (e.g., using more than intended), and psychological (e.g., preoccupation, impaired control) similarities between the compulsive consumption of highly palatable foods and use of addictive drugs [13,14]. Furthermore, confusion exists concerning whether FA more closely represents an emerging form of behavioral addiction, a food-type specific form of chemical dependence, or an emerging subtype of a clinical eating disorder [15]. The lack of a universal definition and concept of FA shows the need for more research on the psychological processes underlying FA.

### 1.2. Prevalence and Correlates of FA

In the recent meta-analysis [16] conducted on 53 studies, the mean prevalence of FA was 16.2%. In a nationally representative study of the US population [17], 15% of participants met the threshold for FA, regardless of body mass index (BMI). In a representative German study with a community sample, the prevalence of FA was 7.9% [18]. FA diagnosis has been observed in 4–10% of non-clinical individuals [19,20]. In postgraduate university students, the prevalence of FA amounted to 4.3% [21]. In a meta-analysis by Pursey et al. [22], a higher prevalence of FA has been noted in patients with overweight/obesity compared to healthy weight individuals (24.9% vs. 11.2%) and in persons with eating disorders compared to individuals with no clinical diagnosis of eating disorders (57.6% vs. 16.2%).

Previous results on the gender-related prevalence of FA have been inconsistent, with some reporting higher FA among women [23,24,25], others reporting that FA is more prevalent in men than in women [26,27], and others reporting no gender differences [4,28]. Meta-analysis has indicated that FA prevalence is double that in women (12.2%) compared to men (6.4%) [22]. Similar to gender, findings regarding the associations of FA with age have been mixed. Although some studies have noted a higher FA prevalence among younger individuals [18,19,29], a meta-analysis by Pursey et al. [22] has shown that adults older than 35 years met the criteria for FA more often than adults younger than 35 years (22.2% vs. 17%).

There are few data on the relationships between personality traits (Big Five) and FA. In a non-clinical sample of Asian university students, lower agreeableness and higher extraversion were correlated with addiction-like eating [30]. In a study conducted among obese patient candidates for bariatric surgery, FA was associated with higher neuroticism, lower conscientiousness, and lower extraversion [31].

As for other psychological variables related to FA, Omar et al. [32] noted that women with FA were significantly more impulsive and more novelty seeking, more harm avoidant, more self-transcendent, less self-directed, and less cooperative than those without FA. Previous studies on the relationship between narcissism and ED [33,34] have suggested that narcissism may predict FA. This relationship may be rooted in low self-esteem and negative ideas about one’s self [35]. Low self-efficacy may be another psychological variable predisposing individuals to symptoms of FA [36,37]. A number of studies have also found a positive association between FA and social anxiety [38] and loneliness [26,39,40].

Regarding the impact on psychological functioning, FA may lead to functional negative consequences, associated distress, and cause a risk to psychological well-being [41]. It is associated with higher levels of depression [16,20,30], anxiety [16,27,30], and psychiatric distress [16,27]. Previous studies have also noted a negative relationship between FA and quality of life, including decreased health quality [20,40,42] and poor sleep [43,44]. While investigating the potential consequences of FA on psychological functioning, the importance of controlling for the effects of relevant predictors should be emphasized to reduce the risk of overestimating the actual impact of FA on functioning [40].

### 1.3. Measurement FA with the YFAS and Its Limitations

The YFAS is the most commonly used scale for measuring FA [45]. It was originally based on substance dependence criteria in the Diagnostic and Statistical Manual of Mental Disorders, Fourth Edition, Text Revision (DSM-IV-TR [46]) and scales used to assess behavioral addictions, such as gambling, exercise, and sex addictions. Subsequent versions are also congruent with the Fifth Edition of the DSM (DSM-5 [47]). All YFAS versions followed similar analytical procedures applied to the development of the scales and the same method for assessing FA risk (separate addiction symptoms and clinical significance items) [29].

There are a few challenging issues with the YFAS that could be addressed with a different analytical approach. Firstly, the items are divided into addiction symptoms and clinical significance indicators. Some psychopathologies are diagnosed in such a way, e.g., schizophrenia which requires the presence of certain symptoms such as delusions (criterion A) and clinically significant impairment (criterion B) [47] (p. 99). However, substance use disorders, according to DSM-5, are not diagnosed with different categories of criteria (symptoms and clinical significance) but with the presence of a certain number of symptoms that are already defined as expressing clinical significance [47]. Additionally, the severity of the disorder is established based on the number of criteria that are met. Accordingly, the existing most commonly used measures of addictive disorders use this approach, e.g., Internet Gaming Disorder Scale [48], pathological gambling measures [49], Problematic Pornography Use Scale [50], and compulsive shopping-buying scales [51]. Typically the importance of incorporating harm caused by the compulsive/addictive behavior is emphasized, and often scales lack properly defined items capturing it.

In the case of the YFAS, items measuring symptoms with a frequency scale are subsequently dichotomized to provide diagnostic thresholds based on the analysis of the relation between scores on the YFAS items and increased risk for broadly defined eating pathology, including binge eating disorder (BED), anorexia nervosa (AN), bulimia nervosa (BN). The YFAS factor analyses are usually conducted on these dichotomized items [29,52]. Such dichotomization has two drawbacks. Firstly, it defines thresholds based on other eating disorders and not FA per se. This likely affects the validity of measurement as items (for the final and subsequent versions of the YFAS) may be selected to measure other non-addictive patterns of disordered eating rather than the phenomenon of FA, e.g., BED or even non-pathological habits of overeating or self-perceived overeating.

This latter phenomenon is well documented in the literature, as women interpret similar overeating behaviors differently, reporting more loss of control than men [53]. It likely affects any diagnosis of an eating disorder involving loss of control as a symptom. Moreover, women tend to have more body image problems related to feeling overweight when not being overweight, which may affect their interpretation of normal eating as overeating [54]. All such instances may be inadequately interpreted as manifestations of FA. However, addictive behavior requires two main criteria to be met: compulsion/loss of control and the negative consequences that it causes. Self-perceived overeating that does not lead to any functional impairments should be excluded as a diagnostic criterion. Consequently, all items representing such notion, and underestimating harm, should be eliminated from scales measuring FA. Moreover, patients with AN may experience a feeling of overeating while effectively starving themselves. Eating disorders are associated with cognitive biases regarding fatness/thinness, dieting, and control of food intake or body weight [55].

In addition, dichotomization narrows down the variance of scores reducing the precision of measurement. Specifically, in the YFAS and the mYFAS clinical significance items were measured as dichotomous answers “yes” or “no,” differently to symptoms’ items which were measured on a frequency scale. This has been modified in the YFAS 2.0 [29] to create a uniform frequency response scale for all items. Addiction can be measured as a continuous phenomenon representing a spectrum, and such an approach to measurement was demonstrated to show advantageous properties for the analytical investigation of addictive behaviors [56]. Most importantly, it enables capturing nuanced differences in addiction risk and its severity (see [57] for a detailed discussion on the dichotomization of measurement and its consequences in the addiction field).

When developing the YFAS and its subsequent versions, clinical significance items measuring a harm component were not included in factor analyses [52]. Moreover, factor analyses for the initial mYFAS validation studies were not conducted altogether [58]. In consequence, there are limited data supporting one general construct of FA as measured by all mYFAS items [42,59]. The current method assumes that high scores on addiction symptoms when appearing with harm indicators are diagnostic of FA.

The practical consequences of the above-described approach to the YFAS scoring are that: (1) measurement and scoring are complex; (2) symptom count scores instead of total scale scores are used for correlational analyses, which is related to a few problems. The most important one is that this score does not represent FA because, according to the scoring method, clinical significance (harm) should also be present. Moreover, because thresholds are based on comparisons with a wide range of eating pathology and factor analyses are conducted on dichotomized scores, this score likely does not represent pure FA but may capture other non-addictive eating pathologies or even non-pathological overeating habits or perceptions. Finally, the variance of scores is substantially limited, affecting any advanced statistical analyses.

### 1.4. Current Study

The objective of this study was two-fold: (1) to test the validity and reliability of the Polish version of the mYFAS among female and male students, including its factorial structure and measurement invariance between genders, as well as criterion validity separately for women and men, and (2) to compare FA between female and male students, including its prevalence, potential risk factors and consequences. To overcome the aforementioned limitations of the YFAS and its modifications, in this study, we modified the response scale in the mYFAS clinical significance items to be the same frequency scale as other symptoms (similarly to the YFAS 2.0). Subsequently, we assumed that all items measure one latent construct of FA without division into symptoms and clinical significance items. This is congruent with (1) the theoretical assumption that one construct representing addictive disorder is measured; (2) the way addictive disorders are diagnosed in DSM-5; (3) the theoretical assumption that items with a Likert-type scale measure continuous variables representing the frequency of particular behaviors characteristic for FA; and (4) harm dimension should permeate to some level most of the items. Apart from improving measurement validity, such assumptions also have considerable practical significance because they (1) simplify measurement and scoring; (2) allow the use of a total score on the mYFAS in more advanced statistical analyses; and (3) have a higher variance of scores, enabling more precise and nuanced analyses of associations with other variables. This way, a simple, valid, and useful measure can be obtained. Our approach assures that the measurement is congruent with the addiction framework and allows for valid differentiation between addictive and non-addictive problematic eating behaviors (such as BED) or non-problematic eating habits or perceptions. A recently developed FA scale used procedures congruent with our approach to measuring this problematic behavior [27].

We formulated the following hypotheses:

**Hypothesis** **1** **(H1).** *The Polish version of the mYFAS will yield a single factor solution representing one construct of FA, and the items will be invariant between genders*.

**Hypothesis** **2** **(H2).** *Women will show higher prevalence rates of FA than men. In addition, our study examined the potential antecedents and consequences of FA.*

Based on the previous studies on the associations between psychological variables and FA [30,31,37,39,40], we hypothesized that:

**Hypothesis** **3** **(H3).** *Narcissism, social anxiety, and loneliness will be positively associated with FA, whereas emotional stability, agreeableness, conscientiousness, self-esteem, and self-efficacy would be negatively associated with FA.*

Since previous studies noted gender differences in the symptoms of addictions (for a review, see [60]), Big Five personality traits [61,62], and other personality traits [63,64], as well as there is some evidence that gender may serve as a moderator of the relationship between psychological variables and symptoms of addictions [65], we hypothesized that:

**Hypothesis** **4** **(H4).** *There will be some differences between genders in terms of the antecedents of FA.*

Regarding the consequences of FA and considering the results of previous studies on predictors of well-being [66,67,68], we expected that:

**Hypothesis** **5** **(H5).** *Higher levels of FA would be associated with lower levels of the general quality of life, health quality, sleep quality, and higher levels of perceived stress, general anxiety, hopelessness, and BMI, even after controlling for age, Big Five personality traits, self-esteem, narcissism, self-efficacy, social anxiety, and loneliness.*

## 2. Materials and Methods

### 2.1. Sample

The sample consisted of 1182 undergraduate students (613 women, 559 men, and 10 persons who did not report their gender) from three major universities in the Pomerania region: University of Gdańsk (*n* = 515, 43.6%), Technological University of Gdańsk (*n* = 470, 39.7%), and Gdańsk University of Physical Education and Sport (*n* = 197, 16.7%). Most students were from first (*n* = 737, 62.3%) and third (*n* = 266, 22.5%) year of study, with similar number of students from second (*n* = 97, 8.2%) and fourth (*n* = 76, 6.4%) year, and minority of fifth (*n* = 3, 0.3%) year students; 3 students (0.3%) did not declare the year of study. Students were from the following courses of study: law (*n* = 289, 24.5%), physical exercise (*n* = 197, 16.7%), administration (*n* = 111, 9.4%), electronics, telecommunications and informatics (*n* = 96, 8.1%), construction (*n* = 93, 7.9%), ocean engineering (*n* = 86, 7.3%), automatics and robotics (*n* = 65, 5.5%), economic analysis (*n* = 60, 5.1%), biomedical engineering (*n* = 56, 4.7%), medical biology (*n* = 54, 4.5%), biology (*n* = 38, 3.2%), German philology (*n* = 18, 1.5%), transport (*n* = 9, 0.8%), Russian studies (*n* = 5, 0.4%), and other (*n* = 5, 0.4%).

Data from 25 participants (2.1%) were removed due to missing data exceeding 30%. The final sample comprised 1157 full-time students (601 women, 546 men, and 10 persons who did not report their gender). The mean age of the participants was 20.33 years (*SD* = 1.68; range: 18–36). The average BMI for those who were sampled was 22.42 kg/m^2^ (*SD* = 3.48; range: 14.28–47.03). Of the 1157 participants, 852 (73.6%) were of normal weight (i.e., BMI from 18.5 to 24.99 kg/m^2^); 94 (8.1%) were underweight (BMI < 18.50 kg/m^2^), and 211 (18.3%) were overweight (BMI ≥ 25 kg/m^2^). Inclusion criteria were undergraduate student status and at least 18 years of age. Exclusion criteria were not being a student or being underaged.

### 2.2. Measures

#### 2.2.1. Food Addiction

We used the modified version of the Yale Food Addiction Scale (mYFAS) [58] to measure FA. The mYFAS consist of 9 out of 25 items of the full version of the YFAS [69] (sample item: “Eating the same amount of food does not reduce negative emotions or increase pleasurable feelings the way it used to.”). The mYFAS was created by choosing one item for each of the seven diagnostic criteria for substance dependence and including two items to assess clinical significance. Respondents are asked about their eating behaviors in the past 12 months. Items are assessed using a 5-point Likert-type scale, with response categories ranging from 1 = “never” to 5 = “4 or more times a week or daily.” The validity and reliability of the scale were assessed with a different approach in previous studies [58].

#### 2.2.2. Personality

To measure the Big Five personality traits (extraversion, emotional stability, agreeableness, conscientiousness, and openness to experience), we used the Polish version of the Ten Item Personality Inventory (TIPI) [70]. Each personality trait is measured with two items, one positively and the other negatively formulated (sample item for extraversion: “I see myself as extraverted, enthusiastic;” for emotional stability: “I see myself as anxious, easily upset;” for agreeableness: “I see myself as critical, quarrelsome;” for conscientiousness: “I see myself as dependable, self-disciplined;” for openness to experience: “I see myself open to new experiences, complex”). Each item is assessed on a 7-point Likert scale (from 1 = “strongly disagree” to 7 = “strongly agree“). The instrument has shown good validity and reliability in previous studies [71,72,73]. In the current study, the Spearman–Brown reliability coefficient was 0.68 for extraversion, 0.56 for emotional stability, 0.29 for agreeableness, 0.65 for conscientiousness, and 0.28 for openness to experience. These values were reasonably similar to those obtained in the original version of the scale, which were 0.68, 0.73, 0.40, 0.50, and 0.45, respectively. As Gosling et al. [70] state, the TIPI has shown good validity, and low estimates of internal consistency result from a very small number of items per construct. The test–retest reliability, which may be considered a less biased measure of reliability in the case of short scales, yielded acceptable correlations between two measurements within a six-week interval, ranging from 0.62 for openness to 0.77 for extraversion [70].

#### 2.2.3. Self-Esteem

Self-esteem was measured with a single item developed based on an item from the World Health Organization Questionnaire of QOL (WHOQOL-BREF) [74]. The item (“How satisfied are you with yourself?”) is answered using a 9-point scale, ranging from 1 = “very dissatisfied” to 9 = “very satisfied.” Previous studies [75,76] supported good validity and reliability. In the previous study, the interclass correlation for test–retest reliability within a 3-week interval between measurements was 0.79 [75].

#### 2.2.4. Perceived Narcissism

Perceived narcissism was measured with the Single Item Narcissism Scale (SINS) [77], which consists of the following statement: “I am a narcissist (Note: The word ‘narcissist’ means egotistical, self-focused, and vain).” In the current study, we used a 9-point Likert-type scale with response options varying from 1 = “No” to 9 = “Yes.” The item demonstrated good validity in previous studies [78].

#### 2.2.5. Self-Efficacy

Self-efficacy was measured with two items based on the General Self-Efficacy Scale (GSES) [79]: “I can usually handle whatever comes my way” and “I can solve most problems if I invest the necessary effort.” These two items have demonstrated the highest content validity, and the criterion validity of the two-item measure has been supported by previous studies [76]. Responses are given using a 9-point Likert-type scale ranging from 1 = “No” to 9 = “Yes.” In the current study, the Spearman–Brown reliability coefficient was 0.81.

#### 2.2.6. Social Anxiety

Social anxiety was measured with a short Polish version of the Liebowitz Social Anxiety Scale [80], based on Dąbkowska’s [81] adaptation. The Polish version consists of 9 out of the 24 items from the original scale, which measure the component of fear experienced in social situations, such as “giving a prepared oral talk to a group.” Items are assessed using a 4-point scale ranging from 0 = “none” to 3 = “severe.” Studies have supported the good validity of this version of the scale [76]. In the current study, Cronbach’s alpha coefficient was 0.83.

#### 2.2.7. Loneliness

Loneliness was measured with the Three-Item Loneliness Scale (TILS) [82]. The TILS consists of three items (sample item: “How often do you feel isolated from others?”), with three response options for each item (1 = “hardly ever,” 2 = “some of the time,” and 3 = “often.” The scale has shown good reliability and validity in previous studies in Polish samples [83]. In the current study, Cronbach’s alpha coefficient was 0.79.

#### 2.2.8. Quality of Life

Three aspects of quality of life were measured in the current study: general quality of life, health quality, and sleep quality. We used three ultra-brief scales developed by Atroszko [71] based on the items from the WHOQOL-BREF [74]. General quality of life was measured with the question: “How would you rate your quality of life?”. General health was measured with the question: “How satisfied are you with your health?”. Sleep quality was measured with the question: “How satisfied are you with your sleep?”. Items are assessed using a 9-point response scale ranging from 1 = “very poor” to 9 = “very good“ for the general quality of life and from 1 = “very dissatisfied“ to 9 = “very satisfied“ for health quality and sleep quality. The scales have shown good validity and reliability in previous studies [84,85].

#### 2.2.9. Perceived Stress

Perceived stress was measured using the Perceived Stress Scale-4 (PSS-4), which is the short form of the PSS-10 [86]. PSS-4 consists of 4 items, 2 positively and 2 negatively formulated (sample item: “In the last month, how often have you felt confident about your ability to handle your personal problems?”) rated on a 5-point Likert scale from 0 = “never” to 4 = “very often.” The Polish version of the PSS-4 has shown good validity and reliability in previous studies [71,87]. In the current study, Cronbach’s alpha coefficient was 0.69.

#### 2.2.10. General Anxiety

General anxiety was measured with a short anxiety scale developed for the Health and Retirement Study [88]. The scale consists of 5 items (sample item: “I had fear of the worst happening”) assessed using a 4-point Likert response format scale, ranging from 1 = “never” to 4 = “most of the time.” Participants were asked to indicate how often they felt in a particular way during the past week. The version of the scale used in this study has shown good validity and reliability [56]. In the current study, Cronbach’s alpha coefficient was 0.87.

#### 2.2.11. Hopelessness

Hopelessness was measured with a Polish version [85] of the Short Hopelessness Scale [88]. The Polish version of the SHS consists of 3 out of 4 items from the original scale (sample item: “The future seems hopeless to me and I can’t believe that things are changing for the better”). Responses are given using a 6-point Likert-type scale ranging from 1 = “I totally disagree” to 5 = “I totally agree.” The scale has shown good validity in previous studies [85]. In the current study, Cronbach’s alpha coefficient was 0.86.

#### 2.2.12. Body Mass Index (BMI)

BMI was measured by asking respondents to report their height and weight and using the following formula: BMI = kg/m^2^.

### 2.3. Procedure

Data were collected using convenience sampling during the winter term in 2016. The research team member (P.A.A.) contacted lecturers via email and asked them to disseminate the survey among the students during their own lectures or classes. Before completing the questionnaires, each student was informed about the purpose of the study, the anonymous and voluntary participation, and the right to withdraw from the study at any point without any consequences. While giving participants sufficient information about the research project and the voluntary participation, all research participants gave their permission to be part of a study by completing the questionnaires and turning them back to the researchers. Completing questionnaires took about 15–20 min. No incentives were given to participants.

Non-probability sampling was used in the present study. However, relatively large, diverse in terms of course fields and years of study, and balanced in terms of gender the sample of students took part in the study, assuring high statistical power and relative generalizability of the results. The advantage of the sampling method was that almost all students from a particular course participated in the study, thus limiting bias from non-responders which in practice is relatively high even in randomly drawn or stratified samples.

The study was approved by the institutional review board at the Institute of Psychology at the University of Gdańsk (approval number 13/2013 obtained on 30 October 2013). The study was part of a multiphase research project on behavioral addictions that consisted of subsequent data collections for the six-year period from 2013 to 2018. All procedures performed in our study were in accordance with the 1964 Helsinki declaration (adopted by the 18th World Medical Association General Assembly, Helsinki, Finland) and its later amendments or comparable ethical standards.

### 2.4. Statistical Analysis

The analysis started with the imputation of missing data using the expectation-maximization (EM) algorithm [89] implemented in Missing Values Analysis within IBM SPSS version 27 [90]. This method was deemed sufficient as the percentage of missing data was small (less than 1%).

In the next step, the sample was randomly divided into two parts (*n*_1_ = 579) and (*n*_2_ = 578), using the Select Cases function implemented in IBM SPSS [90]. Results of the χ^2^ test for gender (*p* = 0.80) and of the *t*-test for age (*p* = 0.25) showed no demographic differences between the two samples. The first sample was used to determine the factor structure of the mYFAS using confirmatory factor analysis (CFA) with the maximum likelihood estimation with robust standard errors (MLR). All nine items were included in the analyses. A single-factor model representing one latent construct of FA was assumed. The second sample was used to test if the findings would replicate. A model with a single latent factor was tested. The following model criteria were employed: χ^2^ divided by degrees of freedom (χ^2^/*df*), Comparative Fit Index (CFI), Tucker–Lewis Index (TLI), Root Mean Squared Error of Approximation (RMSEA), and Standardized Root Mean Square Residual (SRMR). The model fits the data well when: χ^2^/*df* ≤ 3, CFI and TLI ≥ 0.95, RMSEA < 0.06 and SRMR < 0.08 [91,92]. An acceptable model fit is indicated by χ^2^/*df* ≤ 5, CFI and TLI ≥ 0.90, RMSEA < 0.08 and SRMR < 0.1 [93].

Due to low factor loadings of items 1 (“I find myself consuming certain foods even though I am no longer hungry.”) and 2 (“I worry about cutting down on certain foods.”), and poor model fit with item 3 (“I feel sluggish or fatigued from overeating.”), which also showed lower factor loadings than other items, these three items were removed from the scale. The remaining six-item model showed a good fit. Very similar and high (>0.70) item loadings were observed. Based on that, a tau-equivalent model was subsequently tested alongside a congeneric model in the validation subsample. It assumes equal item loadings, which supports the notion that all items measure FA with the same and high precision.

After establishing the structure of the short Polish version of the mYSAS, we performed a multi-group CFA (MGCFA) [94] in the full sample to test the mYFAS measurement invariance (MI) between genders. This method involves running a set of increasingly constrained structural equation models and testing if the differences between these models are significant [95]. We used the congeneric model as a tau-equivalent model is too restrictive in most cases. The following models of MI were tested: (1) configural model, in which the same factor structure is imposed among two groups, with all factor loading and intercept parameters being freely estimated across these groups; (2) metric model, in which the factor loadings between the observed items and the latent variable are set to be equivalent in the two groups; and (3) scalar model, in which both factor loading and intercept parameters are set to be equal across two groups [96,97]. To evaluate the MI of the mYFAS items, CFI, TLI, RMSEA, and SRMR changes (Δ) were investigated. These alternative fit indexes are proved to be more powerful in detecting a lack of invariance in large sample sizes compared to χ^2^, which is overly sensitive to sample size [98,99]. MI was considered established when comparisons of subsequent models showed ΔCFI and ΔTLI < 0.010, ΔRMSEA < 0.015, ΔSRMR < 0.030 (for loading invariance) and SRMR < 0.10 (for intercept invariance) [98,99]. If measurement invariance restriction did not hold for all items (partial invariance)*,* modification indices were inspected to identify the noninvariant items and to refine the structural models [100]. If the majority of parameters are invariant, the partial invariance model results allow us to make meaningful comparisons of structural parameters across the groups [96,97]. CFA and MGCFA were performed using MPlus version 8 [101].

Next, the prevalence of FA based on different approaches to establishing cut-off scores was calculated. First, we calculated the prevalence of FA for nine items using the original cut-offs [58]. To meet the FA threshold, people need to meet the diagnostic threshold for either of the two harm-related questions (responses 3 or 4) and meet the threshold for three or more of the other questions (responses 3 or 4, or only 4, depending on the question). Next, after removing three items from the mYFAS, we used the original cut-offs to calculate the prevalence of FA for six items (two harm-related and four other). Therefore, we retained the most diagnostic items and maintained the number of symptoms that a person needs to meet in order to be diagnosed with FA. This allows us to estimate prevalence when non-diagnostic items were removed. Lastly, we treated all six items as equally diagnostic, and besides calculating the prevalence of FA, we categorized FA as mild (two to three symptoms), moderate (four to five symptoms), or severe (six symptoms), similarly to a recent approach to the mYFAS 2.0 [29]. However, this method should be considered a measurement-derived with the assumption that all items equally and highly contribute to FA rather than symptom-derived, in which each item represents a different symptom. In our six-item version, two items represent clinical significance as defined originally by the authors of the scale. Moreover, three items representing other symptoms were removed as non-diagnostic. However, CFA confirmed that all remaining items equally contribute to the measurement of FA, and likely all are saturated with the “harm” component to a considerable extent.

In the last step of the analyses, the potential predictors and outcomes of FA were tested. Considering the substantial differences between genders in terms of patterns, predictors, and consequences of behavioral addictions [56,102] and taking into account scalar measurement invariance of the mYFAS, we analyzed data separately for men and women. For this aim, a series of hierarchical multiple regression analyses were performed. First, sociodemographic variables, personality traits, self-esteem, and psychosocial factors were consecutively introduced into the model as predictors of FA. Next, the relationships between FA and positive and negative indicators of well-being and BMI were examined, adjusting for age, personality traits, self-esteem, narcissism, self-efficacy, social anxiety, and loneliness. These analyses were analogous to models previously tested in behavioral addictions (shopping, Facebook) [76,103]. Such an approach allows us to compare the relative meaning of potential risk factors of FA and its contribution to potential negative effects on well-being. Detailed justification for these models can be found elsewhere [76,103]. Calculations were performed using IBM SPSS version 27 [90].

## 3. Results

### 3.1. Preliminary Analysis

Table 1 presents the mYFAS items, descriptive statistics, and correlation coefficients separate for men and women. All mYFAS items were positively related except items 1 and 9 in men. Notably, correlations of items 1–3 with other items were weak (*r* between 0.1 and 0.3) or moderate (*r* between 0.3 and 0.5) at most, whereas correlations of items 4–9 were mostly strong (*r* > 0.5).

For men, higher scores than for women were noted for items 5, 7, and 9, whereas women scored higher than men on items 1 and 2. An inspection of descriptive statistics of the mYFAS items, including in particular the values of means, skewness, kurtosis, and correlation coefficients, suggested that items 1–3 might measure a different construct than the remaining items.

### 3.2. Factorial Structure

The results of testing the factorial structure of the nine-item version of the mYFAS are presented in Table 2. The values of model fit criteria (χ^2^/*df* = 7.71, CFI = 0.864, TLI = 0.818, RMSEA = 0.108 (90% CI [0.094, 0.122], SRMR = 0.078) suggested that the model with a single latent factor composed of nine indicators did not fit the data well. Moreover, the standardized factor loadings for items 1–3 were 0.20, 0.37, and 0.51, respectively, being lower than other items in the scale and lower than the recommended threshold of 0.60–0.70 [104,105,106]. Considering these results, as well as values of the descriptive statistics for the mYFAS items (see Table 1), items 1–3 were deleted from the Polish version of the mYFAS. The model with a single latent factor composed of six indicators was then re-examined, indicating a good fit to data (χ^2^/*df* = 1.92, CFI = 0.989, TLI = 0.981, RMSEA = 0.040 (CI 90% [0.006, 0.068], SRMR = 0.021) and high and very similar standardized factor loadings (ranging from 0.72 to 0.81; see Table 2).

To further support the six-item mYFAS structure, CFA was repeated in the second sample (*n* = 578). The model fit the data well: χ^2^/*df* = 2.68, CFI = 0.979, TLI = 0.965; RMSEA = 0.054 (90% CI [0.028, 0.080]), and SRMR = 0.024. All standardized factor loadings exceeded the value of 0.7 (see Table 2). Moreover, due to the similarity of factor loadings in the previous subsample testing, a tau-equivalent model assuming equality of factor loadings was investigated, and showed a good fit (χ^2^/*df* = 2.31, CFI = 0.975, TLI = 0.973; RMSEA = 0.048 (90% CI [0.026, 0.069]), and SRMR = 0.045), with each item loading of 0.77. Cronbach’s alpha coefficient for the six-item mYFAS was 0.89 in the first subsample and 0.87 in the second subsample.

### 3.3. Measurement Invariance

Table 3 presents the results of testing measurement invariance for the mYFAS items between genders. The one-factor model with six indicators fit the data well both in men and women. The results of MGCFA revealed that measurement invariance across genders was entirely supported at the factor-loading level (Table 3). Changes in fit values between the metric and the configural model were all within the acceptable range. For scalar invariance, ΔRMSEA and ΔSRMR were lower than the established cut-offs, whereas ΔCFI (0.015) and ΔTLI (0.01) slightly exceeded the recommended threshold (<0.01). Thus, after the inspection of modification indices, we relaxed the intercept of item 9 to differ across genders [96,97]. Freeing the intercept of item 9 substantially improved the model fit, supporting partial scalar invariance of the mYFAS (see Table 3).

The results of MGCFA suggest that latent mean values obtained from men and women may be compared meaningfully [96]. To compare the latent mean between genders, we constrained the female group’s latent mean to zero, and the male group’s latent mean was free to estimate. To assess latent mean differences, we used the critical ratio (CR) value, obtained by dividing the parameter estimate by standard error. A positive CR implies that the comparison group has a higher latent mean than the reference group. The results of the analysis showed that the latent mean for FA was not significantly different between men and women (CR = 1.82; *p* = 0.069).

### 3.4. Prevalence of Food Addiction

The prevalence of FA based on different approaches to establishing cut-off scores is presented in Table 4. Prevalence was 1.5 times higher among women when estimated based on the original 9-item scale. When items with low factor loadings were removed, the estimates of prevalence based on the original cut-off method and the six-item mYFAS were almost exactly the same for women and men, which was congruent with a lack of differences in the latent mean between genders noted in the current study (see Section 3.3). There were somewhat more men fulfilling cut-offs for FA (mild and moderate) if all six items were treated as equally diagnostic; however, the most severe cases were almost four times more prevalent among women.

### 3.5. Relationships between Food Addiction and Other Variables

Means, standard deviations, percentages, and correlation coefficients of the study variables for both genders separately are presented in Table S1. Among women, FA correlated with all the studied variables except extraversion, whereas among men, FA correlated with all the studies variables except extraversion, agreeableness, and conscientiousness.

#### 3.5.1. Predictors of Food Addiction

The results of regression analysis for FA showed that the predictors explained a total of 15.7% of the variance of FA for men and 11.2% of the variance of FA for women (Table 5). In the final, fourth block of variables, for both genders FA was positively associated with age (men: β = 0.20, *p* < 0.001; women: β = 0.16, *p* < 0.001), extraversion (men: β = 0.09, *p* = 0.046; women: β = 0.12, *p* = 0.012), narcissism (men: β = 0.16, *p* < 0.001; women: β = 0.11, *p* = 0.006), and social anxiety (men: β = 0.12, *p* = 0.008; women: β = 0.12, *p* = 0.016). Moreover, among men, FA was negatively associated with self-efficacy (β = −0.21, *p* < 0.001), whereas among women it was negatively associated with agreeableness (β = −0.11, *p* = 0.018), conscientiousness (β = −0.08, *p* = 0.046), and self-esteem (β = −0.11, *p* = 0.021), and positively with loneliness (β = 0.11, *p* = 0.015).

#### 3.5.2. Relationships between Food Addiction, Well-Being, and BMI

Table 6 and Table 7 present the results of hierarchical regression analysis in which we tested the predictive role of FA on well-being and BMI for men and women separately. After controlling for other variables, FA turned out to be a significant predictor for health quality (men: β = −0.22, *p* < 0.001; women: β = −0.11, *p* = 0.008), general anxiety (men: β = 0.21, *p* < 0.001, women: β = 0.14, *p* < 0.001), and hopelessness (men: β = 0.17, *p* < 0.001; women: β = 0.12, *p* < 0.001) for both genders. Moreover, after adjusting for other variables, FA predicted perceived stress (β = 0.17, *p* < 0.001) among men, and sleep quality (β = −0.14, *p* < 0.001) among women. The explained variance of well-being indicators ranged from 18% for sleep quality to 44% for hopelessness among men and from 12% for health quality to 37% for hopelessness among women. FA was also a significant predictor of BMI (men: β = 0.16, *p* < 0.001; women: β = 0.17, *p* < 0.001), even after adjusting for other variables. The explained variance for BMI was 7.5% for men and 6.5% for women.

## 4. Discussion

The objective of the study was to investigate the validity and reliability of the Polish version of the mYFAS among female and male students, including its factorial structure and measurement invariance between genders, as well as criterion validity separately for women and men. Moreover, the aim of this research was to compare FA between female and male students, including its prevalence, potential risk factors and consequences. To the best of our knowledge, it is the first study to provide a very detailed psychometric analysis of the mYFAS and the first to analyze potential similarities and differences in FA between genders comprehensively.

### 4.1. Factorial Validity

The mYFAS response scale was somewhat modified to evaluate its factorial structure and psychometric properties more adequately. The original structure of the scale did not yield an acceptable factorial solution. The short version of the scale showed a very good model fit, including a tau-equivalent model with the assumption of the equality of loadings on all items. Moreover, scalar measurement invariance allowing for meaningful comparisons between genders was supported (H1 substantiated). Arguably, the removed items also showed the lowest content validity as they measure eating patterns that are not always pathological and can be relatively frequent in the general population. In fact, they were originally designed this way by authors to assess behaviors that could plausibly occur occasionally in non-problem eaters. However, while all items that originally were rated on the frequency scale were described this way, CFA showed that the three first items, in fact, very poorly capture problematic eating and may not be diagnostic at all. Importantly, women scored significantly higher on the first two items (removed), while men scored significantly higher on items 5, 7, and 9, measuring clinically relevant symptoms such as withdrawal or functional impairments.

### 4.2. Prevalence of FA

Consequently, the prevalence analyses showed that FA rates were 1.5 times higher among female students than male students when estimated using the original scoring method and based on the original 9-item scale (see Table 4). This finding was congruent with a meta-analysis of prevalence based on the YFAS finding higher rates among women [22]. However, H2 was only partially supported; when items with low factor loadings were removed, the estimates of prevalence based on the original cut-off method and six-item scale were almost exactly the same for women (2.3%) and men (2.6%). These were also very similar to the ones reported with a recently developed FA scale, which used procedures congruent with our approach to measuring this problematic behavior [27]. There were somewhat more men fulfilling cut-offs for FA (mild and moderate) if all six items were treated as equally diagnostic; however, the most severe cases were almost four times more prevalent among women. Usage of non-diagnostic items which may measure non-pathological eating behaviors likely contributes to the overestimation of FA among women and exaggerates gender differences. This conclusion is consistent with findings of generally higher obesity rates among men rather than women in developed countries [107], as well as with results that men may underestimate or women may overestimate problematic eating behaviors [53].

### 4.3. Potential Antecedents and Consequences of FA

Subsequently, the short version of the mYFAS was used in a series of regression analyses in which FA was a potential outcome and a potential cause of deteriorated well-being. These analyses were conducted separately for female and male students because a considerable body of evidence has suggested that addictive disorders function differently between genders (for an overview, see [57]). A recent study revealed that, similarly to substance use disorders, behavioral addictions demonstrate clear patterns of different profiles between women and men [56]. Moreover, another research using the YFAS showed that FA is among the addictive behaviors associated with the female gender [22,57]. As expected, there are some common antecedents of FA between men and women, including social anxiety, narcissism, extraversion, and age (H3 partially substantiated). In addition, in the penultimate step of regression analyses, low emotional stability showed a similar significant relationship to FA in both genders, which was somewhat diluted most likely by social anxiety added in the last step of the analyses. These results are congruent with previous findings showing that emotionally unstable, anxious and narcissistic individuals generally have higher risks for addictive behaviors [56] and that addictions tend to progress with age. High extraversion seems to be a distinct FA risk factor, common to men and women. Previous analyses have demonstrated that it is rather characteristic of the female profile of addictions [56]. Similarly to social networks use disorder (SNUD) [76], the discrepancy between extraversion-related social needs and high social anxiety may create tension driving unhealthy coping mechanisms related to food.

Notably, however, there were differences in potential specific antecedents of FA between female and male students (H4 substantiated). Among women, low agreeableness, conscientiousness, and self-esteem, together with high loneliness, were significantly related to FA. On the other hand, among men, none of these were related to FA in the final model; instead, self-efficacy stood out as showing the relatively strongest association. While self-esteem was negatively related to FA among men in the penultimate step of the analysis, self-efficacy likely explained this covariance when it was added in the last step. This was not the case among women. These results suggest a clear distinction between socially oriented communion variables and an agentic variable [108], differentiating FA risks between genders. Women who show impairments in social functioning due to being disagreeable, likely quarrelsome, and impulsive (of which low conscientiousness may be to some extent indicative) may use food as a coping mechanism with loneliness and social rejection, including fear of social rejection (social anxiety) [109,110,111,112].

On the other hand, men addicted to food may use eating as a coping mechanism with their sense of low generalized self-efficacy and hopelessness. These findings are congruent with previous analyzes showing that the female profile of addictions includes more socially oriented behaviors, particularly those associated with social perception by others, such as SNUD, study addiction, and shopping addiction [56]. Women tend to addict more to behaviors that focus around creating and maintaining the social image of an attractive (social networking sites), intelligent and diligent (study), and wealthy/affluent (shopping) person. On the other hand, men are generally more prone to addictive behaviors and substances. It is driven by a sense of hopelessness and helplessness related to real-life failures in agentic domains than strivings for social self-presentation. Consequently, their risky behaviors more often turn into a serious addiction, considerably negatively affecting their psychosocial functioning. This does not mean that men do not strive for a positive social image via social networking sites or other means, nor that women do not react negatively to failures in agentic domains. In fact, previous analyzes have found that the number of women in the full-blown addiction profile is not trivial and is much more similar to men than in other profiles [56]. More studies are required to determine how gender differences manifest at different levels of FA risk. It is likely that men and women fulfilling the criteria for clinical levels of FA are more similar than on the other levels of addictive behaviors and their risk related to food consumption (for an overview of emerging theories on gender differences in behavioral addictions, see [57]).

In both genders, FA showed significant zero-order correlations with all indicators of deteriorated well-being and health (general quality of life, health quality, sleep quality, perceived stress, general anxiety, hopelessness, and BMI). This supports the criterion validity of the short version of the mYFAS. However, both for women and men, it was related only to lower health quality and higher general anxiety, hopelessness and BMI above and beyond relevant personality traits and psychosocial variables (H5 partially substantiated). Moreover, there was a statistically significant relationship with perceived stress above and beyond other variables in the model among men and sleep quality among women. The mean difference in betas from the last step of regression analyses across all well-being indicators (0.12) showed that generally, the associations were relatively stronger for men, particularly in terms of health quality and perceived stress.

Consistent with previous research [22,53,54], four findings from the current study taken together suggest that level of problematic eating behaviors among women may be overestimated, and the gravity of problems among men can be underestimated if not measured properly: (1) higher scores of female students on items removed from the scale due to low factor loadings, i.e., likely measuring common non-pathological eating habits or perceptions; (2) higher scores on clinical symptoms such as withdrawal and functional impairments obtained by male students; (3) somewhat stronger association of FA with deteriorated well-being among male students; and (4) more severe cases identified with cut-off score among female students.

There are two most important theoretical and practical implications of the current study. First is that the measurement of FA can be simplified, and at the same time, it may improve its validity and usefulness, including the availability for advanced and nuanced statistical analyses. The second is that there might be no differences in the prevalence of FA between genders; however, there are different potential risk factors of FA among women and men, and the potential severity disparities in the disorder need further clarification.

This has crucial importance since, thus far, most research on FA has tended to analyze both genders together. Such practice clearly creates more confusion and bias in the way FA is understood. While there are some common risk factors for all addictions (e.g., neuroticism), women and men clearly differ in how they become addicted, how addictions develop and are maintained, in relapse risks and treatment outcomes [60,113,114]. Similar findings indicating the necessity of separate analysis of risk factors for different genders emerge from research on other addictive behaviors. While it may complicate research, it seems indispensable to analyze addictive behaviors separately for women and men.

### 4.4. Strengths and Limitations

In terms of strengths, to the best of our knowledge, this is the first study to investigate in detail the psychometric properties of the mYFAS with a different theoretical and analytical approach. Commonly used valid and reliable psychological questionnaires were applied. A relatively large, diverse and balanced in terms of gender sample of students took part in the study, assuring high statistical power and relative generalizability of the results. It may lay a foundation for future developments of the YFAS and diagnostic criteria for FA, as well as studies on gender differences related to this problematic behavior.

Regarding the limitations of the present study, the sample was not representative of the general population of undergraduate students. Moreover, all the data collected were self-reported, which is related to typical weaknesses of such data, e.g., common method bias. The data were cross-sectional, which precludes any conclusions about causal relationships among the variables. In addition, the data can be considered relatively old since they were gathered in 2016. Nonetheless, the discussed limitations did not seem to prevent identifying the most important and expected effects.

## 5. Conclusions

It can be concluded that the short six-item version of the mYFAS is a valid and reliable measure of FA risk, invariant between genders, and therefore allowing for meaningful comparisons between men and women. It is the first study to examine in detail the theoretical implications of the psychometric properties of the mYFAS analyzed with a different scoring method. It showed that the original version of the scale may be problematic due to the low content, factorial validity, and clinical validity of the first three items. Measurement of FA can be considerably simplified, and at the same time, its validity and usefulness for advanced and nuanced statistical analyses may be improved.

FA may show similar prevalence rates between genders among undergraduate students. The higher prevalence and seemingly higher severity of FA among women found in previous studies may stem from using non-diagnostic criteria/items used to measure its risk. These may overestimate disordered eating behaviors due to the tendency of women to perceive common non-pathological eating habits or perceptions as problematic. It is likely related to cognitive biases driven by cultural standards of a slim body image. At the same time, addictive eating among men and its consequences may be systematically neglected as more studies are conducted on women.

FA has common risk factors among women and men (social anxiety, extraversion, narcissism and age). Importantly, however, there are clear differences in specific risk factors. Communal variables such as low agreeableness and high loneliness together with low self-esteem and conscientiousness are significantly related to FA among women. On the other hand, the agentic trait of low self-efficacy is associated with FA among men. This has crucial importance since, thus far, most research on FA has tended to analyze both genders together. Such practice clearly creates more confusion and bias in the way FA is understood.

Moreover, in both genders, the six-item mYFAS was related to all indicators of deteriorated well-being and health (general quality of life, health quality, sleep quality, perceived stress, general anxiety, hopelessness, and BMI), and it was related to most of the indicators above and beyond other personality traits and variables previously found to be consistently associated with deteriorated psychosocial functioning (high neuroticism, social anxiety and loneliness, and low conscientiousness, self-esteem and self-efficacy). The associations tended to be somewhat stronger among men.

While it may complicate research, future studies should analyze FA and likely most of the addictive behaviors separately for men and women. More representative samples from the general population, and longitudinal studies, including detailed analyses of common and specific risk factors, clinical manifestations, and developmental trajectories, are warranted. Prevention and treatment programs accounting for gender differences in FA are highly needed. More research on FA among men is needed. More advanced analyses of the psychometric properties of FA scales may allow for the development of valid criteria for diagnosing FA and measures based on them.

## Figures and Tables

**Table 1:** Descriptive statistics and bivariate correlations for the mYFAS items among men (above the diagonal; *n* = 546) and women (below the diagonal; *n* = 601).

mYFAS Items	(1)	(2)	(3)	(4)	(5)	(6)	(7)	(8)	(9)
(1) I find myself consuming certain foods even though I am no longer hungry.	**1**	0.33 ***	0.29 ***	0.16 ***	0.11 *	0.14 ***	0.11 *	0.12 **	0.07
(2) I worry about cutting down on certain foods.	0.44 ***	**1**	0.34 ***	0.37 ***	0.29 ***	0.34 ***	0.28 ***	0.35 ***	0.28 ***
(3) I feel sluggish or fatigued from overeating.	0.39 ***	0.43 ***	**1**	0.46 ***	0.39 ***	0.38 ***	0.38 ***	0.32 ***	0.34 ***
(4) I have spent time dealing with negative feelings from overeating certain foods, instead of spending time in important activities such as time with family, friends, work, or recreation.	0.25 ***	0.31 ***	0.55 ***	**1**	0.68 ***	0.63 ***	0.65 ***	0.62 ***	0.59 ***
(5) I have had physical withdrawal symptoms such as agitation and anxiety when I cut down on certain food. (Do NOT include caffeinated drinks: coffee, tea, cola, energy drinks, etc.)	0.15 ***	0.25 ***	0.35 ***	0.55 ***	**1**	0.64 ***	0.59 ***	0.66 ***	0.62 ***
(6) I kept consuming the same types or amounts of food despite significant emotional and/or physical problems related to my eating.	0.29 ***	0.37 ***	0.41 ***	0.55 ***	0.60 ***	**1**	0.63 ***	0.62 ***	0.61 ***
(7) Eating the same amount of food does not reduce negative emotions or increase pleasurable feelings the way it used to.	0.22 ***	0.28 ***	0.39 ***	0.49 ***	0.56 ***	0.57 ***	**1**	0.56 ***	0.52 ***
(8) My behavior with respect to food and eating causes me significant distress.	0.23 ***	0.35 ***	0.33 ***	0.48 ***	0.56 ***	0.58 ***	0.57 ***	**1**	0.71 ***
(9) Issues related to food and eating decrease my ability to function effectively (daily routine, job/school, social or family activities, health difficulties).	0.13 **	0.20 ***	0.30 ***	0.43 ***	0.52 ***	0.47 ***	0.52 ***	0.59 ***	**1**
Men									
*M*	2.81	2.34	2.27	1.71	1.58	1.73	1.75	1.65	1.67
*SD*	1.20	1.24	1.12	1.02	0.97	1.07	1.07	1.04	1.08
Skewness	0.17	0.45	0.49	1.32	1.60	1.32	1.32	1.52	1.54
Kurtosis	−0.87	−0.89	−0.67	0.78	1.62	0.73	0.78	1.33	1.33
Women									
*M*	3.17	3.06	2.38	1.68	1.45	1.68	1.61	1.71	1.42
*SD*	1.27	1.49	1.28	1.09	0.93	1.08	1.03	1.11	0.91
Skewness	−0.15	−0.08	0.64	1.64	2.37	1.52	1.76	1.53	2.33
Kurtosis	−1.05	−1.42	−0.63	1.80	5.25	1.31	2.24	1.41	4.80
T-test	−4.96 ***	−8.89 ***	−1.61	0.43	2.43 *	0.86	2.19 *	−0.91	4.18 ***

Note. * *p* < 0.05; ** *p* < 0.01; *** *p* < 0.001; *M* = mean; *SD* = standard deviation. For readability purposes, significant correlation coefficients were bolded. Points for the mYFAS items range from 1 to 5.

**Table 2:** Standardized item factor loadings and standard errors (in parentheses) for all models.

Item	mYFAS Version		
	Nine-Item (*n*_1_ = 579)	Six-Item (*n*_1_ = 579)	Six-Item (*n*_2_ = 578)
1	0.20 (0.05)	–	–
2	0.37 (0.04)	–	–
3	0.51 (0.04)	–	–
4	0.76 (0.03)	0.74 (0.03)	0.73 (0.03)
5	0.78 (0.03)	0.81 (0.03)	0.77 (0.03)
6	0.80 (0.03)	0.79 (0.03)	0.76 (0.03)
7	0.74 (0.03)	0.74 (0.03)	0.74 (0.04)
8	0.75 (0.03)	0.76 (0.03)	0.80 (0.03)
9	0.71 (0.04)	0.72 (0.04)	0.74 (0.03)

**Table 3:** Goodness-of-fit indices and model comparisons for measurement invariance models.

Model	S-B χ^2^	*df*	CFI	TLI	RMSEA (90% CI)	SRMR	Model Comparison	ΔCFI	ΔTLI	ΔRMSEA	ΔSRMR
Men	36.47	9	0.966	0.943	0.075 (0.050–0.101)	0.027					
Women	15.72	9	0.990	0.984	0.035 (0.000–0.064)	0.022					
(A) Configural	51.08	18	0.978	0.963	0.057 (0.039–0.075)	0.024					
(B) Metric	62.88	23	0.973	0.965	0.055 (0.039–0.071)	0.040	B vs. A	−0.005	0.002	−0.002	0.016
(C) Scalar	90.79	28	0.958	0.955	0.063 (0.048–0.077)	0.039	C vs. B	−0.015	−0.010	0.008	−0.001
(D) Partial scalar *	78.22	27	0.966	0.962	0.058 (0.043–0.073)	0.041	D vs. B	−0.007	−0.003	0.003	0.001

Note. * Free intercept of item 9. S-B χ^2^ = Satorra-Bentler scaled χ^2^; *df* = degrees of freedom. CFI = Comparative Fit Index; TLI = Tucker–Lewis Index, RMSEA = Root Mean Squared Error of Approximation, SRMR = Standardised Root Mean Residual, Δ = change relative to the preceding model. *N* = 1147.

**Table 4:** Prevalence (percentage of the participants fulfilling diagnostic criteria) of food addiction among men and women based on different approaches to establishing cut-off scores.

	Original Method Based on 9 Items	Original Method Based on 6 Items	All 6 Items Treated as Equally Diagnostic		
			Mild 2–3	Moderate 4–5	Severe 6
Men	3.7	2.6	13.1 (9.6)	3.5 (3.1)	0.4
Women	5.5	2.3	11.0. (7.8)	3.2 (1.6)	1.5

Note. For the method with all six items treated as equally diagnostic, clinical significance items represent one symptom; therefore, this should not be considered a symptom-based but rather a measurement-based cut-off analysis. Moreover, for this method, outside parenthesis is an estimate of “at least this category or more severe,” and inside parenthesis is “exactly this category.” For example, 13.1% of men fulfill the criteria for at least mild FA, and 9.6% of men fulfill the criteria only for mild FA and not more severe FA.

**Table 5:** Hierarchical multiple regression analysis predicting food addiction among men (*n* = 546) and women (*n* = 601).

	Food Addiction: Men		Food Addiction: Women	
Predictor	**β**	∆*R*^2^	β	∆*R*^2^
Step 1		0.044 ***		0.013 **
Age	0.21 ***		0.12 **	
Step 2		0.047 ***		0.069 ***
Age	0.20 ***		0.13 **	
Extraversion	0.02		0.03	
Agreeableness	−0.02		**−0** .11 **	
Conscientiousness	−0.06		**−0** .13 **	
Emotional stability	**−0** .14 **		**−0** .12 **	
Openness	**−0** .13 **		**−0** .10 *	
Step 3		0.026 ***		0.025 ***
Age	0.20 ***		0.15 ***	
Extraversion	0.04		0.05	
Agreeableness	0.05		−0.07	
Conscientiousness	−0.04		**−0** .09 *	
Emotional stability	**−0** .12 **		**−0** .09 *	
Openness	**−0** .13 **		−0.08	
Self-esteem	**−0** .11 *		**−0** .14 **	
Narcissism	0.15 ***		0.12 **	
Step 4		0.057 ***		0.020 **
Age	0.20 ***		0.16 ***	
Extraversion	0.09 *		0.12 *	
Agreeableness	0.02		**−0** .11 *	
Conscientiousness	−0.01		**−0** .08 *	
Emotional stability	−0.06		–0.06	
Openness	−0.06		−0.07	
Self-esteem	−0.03		**−0** .11 *	
Narcissism	0.16 ***		0.11 **	
Self-efficacy	**−0** .21 ***		0.04	
Social anxiety	0.12 **		0.12 *	
Loneliness	0.04		0.11 *	
Total R^2^		0.157		0.112

Note. * *p* < 0.05, ** *p* < 0.01, *** *p* < 0.001. Gender: 0 = female, 1 = male. β *=* standardized regression coefficient; ∆*R*^2^ = change in *R*^2^ value between the steps. Numbers in bold indicate significant *p*-values for β.

**Table 6:** Results of hierarchical multiple regression analyses: Food addiction, age, personality, self-esteem, narcissism, self-efficacy, social anxiety, and loneliness as predictors of quality of life among men (*n* = 546) and women (*n* = 601).

	Men						Women					
	General Quality of Life		Health Quality		Sleep Quality		General Quality of Life		Health Quality		Sleep Quality	
Predictor	**β**	**∆*R*^2^**	**β**	**∆*R*^2^**	**β**	**∆*R*^2^**	**β**	**∆*R*^2^**	**β**	**∆*R*^2^**	**β**	**∆*R*^2^**
Step 1		0.037 ***		0.089 ***		0.024 ***		0.013 **		0.031 ***		0.027 ***
Food addiction	**−0** .19 ***		**−0** .30 ***		**−0** .16 ***		**−0** .11 **		**−0** .18 ***		**−0** .17 ***	
Step 2		0.000		0.001		0.001		0.002		0.002		0.015 **
Food addiction	**−0** .19 ***		**−0** .29 ***		**−0** .16 ***		**−0** .12 **		**−0** .17 ***		**−0** .18 ***	
Age	−0.02		−0.04		0.03		0.04		−0.04		0.13 **	
Step 3		0.139 ***		0.058 ***		0.068 ***		0.124 ***		0.026 **		0.012
Food addiction	**−0** .13 **		**−0** .25 ***		**−0** .13 **		−0.06		**−0** .13 **		**−0** .17 ***	
Age	−0.02		−0.05		0.03		0.00		−0.07		0.11 **	
Extraversion	0.24 ***		0.15 ***		0.13 **		0.25 ***		0.04		0.02	
Agreeableness	0.07		0.05		−0.01		0.02		0.05		−0.01	
Conscientiousness	0.10 *		0.10 *		0.06		0.12 **		0.07		−0.02	
Emotional stability	0.16 ***		0.10 *		0.21 ***		0.15 ***		0.10 *		0.11 *	
Openness	0.06		0.02		−0.06		0.03		−0.02		−0.02	
Step 4		0.137 ***		0.074 ***		0.071 ***		0.124 ***		0.073 ***		0.077 ***
Food addiction	**−0** .09 *		**−0** .22 ***		−0.10 *		−0.01		**−0** .10 *		**−0** .13 **	
Age	−0.04		−0.06		0.01		−0.05		**−0** .11 **		0.07	
Extraversion	0.13 **		0.06		0.05		0.16 ***		−0.03		−0.04	
Agreeableness	0.04		0.01		−0.04		0.03		0.05		0.01	
Conscientiousness	0.00		0.03		−0.01		0.04		0.01		−0.08	
Emotional stability	0.08 *		0.04		0.16 ***		0.06		0.04		0.04	
Openness	0.03		0.00		−0.08		−0.02		−0.06		−0.06	
Self-esteem	0.43 ***		0.32 ***		0.31 ***		0.41 ***		0.32 ***		0.32 ***	
Narcissism	0.01		−0.03		0.00		0.01		−0.02		0.03	
Step 5		0.087 ***		0.001		0.016 *		0.040 ***		0.007		0.003
Food addiction	−0.02		**−0** .22 ***		−0.08		−0.01		**−0** .11 **		**−0** .14 ***	
Age	−0.07 *		−0.06		0.00		−0.06		**−0** .10 *		0.08	
Extraversion	0.07		0.06		0.01		0.13 **		0.01		−0.02	
Agreeableness	0.06		0.00		−0.03		0.03		0.02		−0.01	
Conscientiousness	−0.04		0.03		−0.02		0.02		0.01		−0.08	
Emotional stability	0.01		0.04		0.13 **		0.02		0.05		0.05	
Openness	−0.04		0.00		−0.08		−0.05		−0.05		−0.06	
Self-esteem	0.32 ***		0.31 ***		0.26 ***		0.33 ***		0.32 ***		0.33 ***	
Narcissism	−0.01		−0.04		0.00		0.00		−0.02		0.03	
Self-efficacy	0.28 ***		0.04		0.01		0.21 ***		0.04		0.02	
Social anxiety	−0.05		0.02		−0.03		0.07		0.08		0.02	
Loneliness	**−0** .14 ***		−0.01		**−0** .13 **		**−0** .12 **		0.05		0.07	
Total R^2^		0.400		0.207		0.180		0.302		0.121		0.135

Note. * *p* < 0.05, ** *p* < 0.01, *** *p* < 0.001. β *=* standardized regression coefficient; ∆*R*^2^ = change in *R*^2^ value between the steps. Numbers in bold indicate significant *p*-values for β.

**Table 7:** Results of hierarchical multiple regression analyses: Food addiction, age, personality, self-esteem, narcissism, self-efficacy, social anxiety, and loneliness as predictors of perceived stress, general anxiety, hopelessness, and BMI for men (*n* = 546) and women (*n* = 601).

	Men								Women							
	Perceived Stress		General Anxiety		Hopelessness		BMI		Perceived Stress		General Anxiety		Hopelessness		BMI	
Predictor	**β**	**∆*R*^2^**	**β**	**∆*R*^2^**	**β**	**∆*R*^2^**	**β**	**∆*R*^2^**	**β**	**∆*R*^2^**	**β**	**∆*R*^2^**	**β**	**∆*R*^2^**	**β**	**∆*R*^2^**
Step 1		0.089 ***		0.123 ***		0.108 ***		0.038 ***		0.028 ***		0.044 ***		0.056 ***		0.039 ***
Food addiction	0.30 ***		0.35 ***		0.33 ***		0.20 ***		0.17 ***		0.21 ***		0.24 ***		0.20 ***	
Step 2		0.008 *		0.011 **		0.005		0.009 *		0.013 **		0.048 ***		0.015 **		0.001
Food addiction	0.32 ***		0.37 ***		0.34 ***		0.17 ***		0.18 ***		0.24 ***		0.25 ***		0.19 ***	
Age	−0.09 *		−0.11 **		−0.07		0.10 *		−0.11 **		−0.22 ***		−0.13 **		0.03	
Step 3		0.111 ***		0.126 ***		0.160 ***		0.020 *		0.146 ***		0.081 ***		0.125 ***		0.019 *
Food addiction	0.26 ***		0.30 ***		0.27 ***		0.15 ***		0.11 **		0.19 ***		0.18 ***		0.17 ***	
Age	−0.08 *		−0.10 **		−0.07		0.10 *		−0.06		−0.18 ***		−0.09 *		0.03	
Extraversion	−0.14 ***		−0.12 **		−0.21 ***		0.12 *		0.20 ***		−0.17 ***		−0.19 ***		0.04	
Agreeableness	0.07		−0.02		0.02		0.00		0.08 *		0.07		−0.01		−0.10 *	
Conscient.	−0.16 ***		−0.06		−0.17 ***		−0.05		−0.13 ***		−0.08		−0.18 ***		−0.10 *	
Emo. stability	−0.23 ***		−0.30 ***		−0.14 ***		−0.06		−0.27 ***		−0.18 ***		−0.12 **		0.05	
Openness	−0.05		−0.05		−0.17 ***		−0.09		0.00		−0.05		−0.09 *		−0.04	
Step 4		0.086 ***		0.051 ***		0.064 ***		0.002		0.121 ***		0.035 ***		0.150 ***		0.005
Food addiction	0.23 ***		0.28 ***		0.26 ***		0.16 ***		0.07		0.16 ***		0.13 ***		0.16 ***	
Age	−0.07		−0.09 *		−0.06		0.10 *		−0.01		−0.16 ***		−0.03		0.04	
Extraversion	−0.05		−0.05		−0.13 **		0.12 **		−0.11 **		−0.12 **		−0.10 *		0.05	
Agreeableness	0.08		0.01		0.02		−0.02		0.08 *		0.08		0.00		−0.11 *	
Conscient.	−0.08 *		0.00		−0.10 **		−0.04		−0.06		−0.03		−0.10 **		−0.09 *	
Emo. stability	−0.17 ***		−0.25 ***		−0.09 *		−0.06		−0.19 ***		−0.13 **		−0.02		0.07	
Openness	−0.03		−0.03		−0.15 ***		−0.08		0.05		−0.02		−0.03		−0.03	
Self-esteem	−0.34 ***		−0.26 ***		−0.28 ***		−0.02		−0.40 ***		−0.22 ***		−0.45 ***		−0.08	
Narcissism	−0.03		0.00		−0.08 *		−0.05		0.00		0.02		0.02		−0.02	
Step 5		0.062 ***		0.096 ***		0.106 ***		0.006		0.041 ***		0.043 ***		0.026 ***		0.001
Food addiction	0.17 ***		0.21 ***		0.17 ***		0.16 ***		0.05		0.14 ***		0.12 ***		0.17 ***	
Age	−0.04		−0.07		−0.02		0.10 *		0.01		−0.14 ***		−0.01		0.04	
Extraversion	0.02		0.06		−0.06		0.11 *		−0.04		−0.03		−0.03		0.04	
Agreeableness	0.06		−0.03		−0.01		−0.01		0.06		0.04		−0.02		−0.10 *	
Conscient.	−0.05		0.03		−0.06		−0.04		−0.02		−0.01		−0.07		−0.09 *	
Emo. stability	−0.11 **		−0.19 ***		−0.01		−0.06		−0.13 ***		−0.07		0.02		0.07	
Openness	0.02		0.02		−0.08 *		−0.08		0.10 **		0.01		0.01		−0.03	
Self-esteem	−0.24 ***		−0.17 ***		−0.16 ***		−0.01		−0.32 ***		−0.14 **		−0.39 ***		−0.09	
Narcissism	−0.02		0.01		−0.06		−0.05		0.01		0.02		0.03		−0.02	
Self-efficacy	−0.13 **		−0.09 *		−0.29 ***		−0.06		−0.19 ***		−0.10 *		−0.14 ***		0.00	
Social anxiety	0.13 **		0.25 ***		0.10 **		−0.07		0.08		0.06		0.09 *		−0.03	
Loneliness	0.17 ***		0.16 ***		0.15 ***		0.00		0.09 *		0.20 ***		0.05		−0.01	
Total R^2^		0.341		0.407		0.443		0.075		0.336		0.252		0.373		0.065

Note. * *p* < 0.05, ** *p* < 0.01, *** *p* < 0.001. β *=* standardized regression coefficient; ∆*R*^2^ = change in *R*^2^ value between the steps. Numbers in bold indicate significant *p*-values for β. Conscient. = conscientiousness; emo. stability = emotional stability.

## Data Availability

The data presented in this study are available on request from the corresponding author [P.A.A.].

## Supplementary Materials

The following supporting information can be downloaded at: https://www.mdpi.com/article/10.3390/nu14194041/s1, Table S1: Descriptive statistics and correlations between the study variables among men (above the diagonal; *n* = 546) and women (below the diagonal; *n* = 601).
