# Supplementary material for: The Modified Yale Food Addiction Scale May Be Simplified and Diagnostically Improved: The Same Prevalence but Different Severity and Risk Factors of Food Addiction among Female and Male Students
Source: Nutrients. 2022 Sep 28;14(19):4041. doi: 10.3390/nu14194041 (PMC9573175; doi:10.3390/nu14194041)
Supplement: Supplementary file 1 [file nutrients-14-04041-s001.zip › nutrients-1934720-supplementary.pdf]

Supplementary Materials

Table S1. Descriptive statistics and correlations between the study variables among men (above the diagonal; *n* = 546) and women (below the diagonal; *n* = 601).

Variables	(1)	(2)	(3)	(4)	(5)	(6)	(7)	(8)	(9)	(10)	(11)	(12)	(13)	(14)	(15)	(16)	(17)	(18)	(19)
(1) Food addiction[a]	1	**.21*** **	−.04	−.09	−.08	**−.17*** **	**−.15*** **	**−.15*** **	**.15*** **	**−.28*** **	**.21*** **	**.14*** **	**−.19*** **	**−.30*** **	**−.16*** **	**.30*** **	**.35*** **	**.33*** **	**.20*** **
(2) Age	**.12** **	1	−.02	−.02	.06	−.02	**−.08* **	.02	.03	.00	.03	**−.11* **	−.06	**−.10* **	.00	−.03	−.03	.00	**.14** **
(3) Extraversion	−.04	.04	1	.08	**.10* **	**.09* **	**.31*** **	**.34*** **	.06	**.23*** **	**−.36*** **	**−.31*** **	**.30*** **	**.19*** **	**.14** **	**−.20*** **	**−.18*** **	**−.30*** **	.07
(4) Agreeableness	**−.16*** **	−.04	.04	1	.06	**.40*** **	**.12** **	**.22*** **	**−.37*** **	**.11* **	.02	**−.12** **	**.18*** **	**.13** **	**.09* **	−.08	**−.18*** **	**−.11* **	−.05
(5) Conscientiousness	**−.16*** **	**.10* **	**.09* **	.05	1	**.13** **	.07	**.30*** **	.01	**.24*** **	**−.17*** **	**−.16*** **	**.16*** **	**.15*** **	**.11* **	**−.23*** **	**−.14*** **	**−.24*** **	−.05
(6) Emotional stability	**−.17*** **	**.10* **	**.12** **	**.38*** **	**.22*** **	1	.05	**.29*** **	**−.16*** **	**.27*** **	**−.15*** **	**−.25*** **	**.25*** **	**.19*** **	**.25*** **	**−.28*** **	**−.37*** **	**−.23*** **	**−.09* **
(7) Openness	**−.10* **	−.05	**.40*** **	−.03	**.11* **	.02	1	**.20*** **	.06	**.31*** **	**−.29*** **	**−.09* **	**.18*** **	**.13** **	.01	**−.14*** **	**−.14*** **	**−.29*** **	**−.09* **
(8) Self-esteem	**−.18*** **	**.16*** **	**.31*** **	**.10* **	**.30*** **	**.31*** **	**.24*** **	1	−.01	**.38*** **	**−.28*** **	**−.43*** **	**.52*** **	**.39*** **	**.36*** **	**−.45*** **	**−.40*** **	**−.44*** **	−.05
(9) Narcissism	**.14*** **	−.01	.04	**−.32*** **	−.05	−.08	.05	.06	1	.07	−.06	.05	−.02	−.08	−.03	−.01	.08	−.05	−.01
(10) Self-efficacy	**−.11** **	**.11** **	**.33*** **	**.10* **	**.28*** **	**.29*** **	**.34*** **	**.48*** **	.07	1	**−.34*** **	**−.34*** **	**.48*** **	**.24*** **	**.19*** **	**−.40*** **	**−.38*** **	**−.54*** **	**−.11** **
(11) Social anxiety	**.14*** **	**−.11** **	**−.47*** **	**.10* **	**−.21*** **	**−.18*** **	**−.37*** **	**−.34*** **	**−.08* **	**−.42*** **	1	**.35*** **	**−.30*** **	**−.16*** **	**−.17*** **	**.34*** **	**.42*** **	**.39*** **	−.01
(12) Loneliness	**.17*** **	**−.11** **	**−.39*** **	.02	**−.19*** **	**−.27*** **	**−.20*** **	**−.40*** **	−.01	**−.33*** **	**.38*** **	1	**−.41*** **	**−.21*** **	**−.29*** **	**.41*** **	**.42*** **	**.41*** **	.01
(13) Quality of life	**−.11** **	.03	**.29*** **	**.10* **	**.18*** **	**.22*** **	**.15*** **	**.48*** **	.02	**.40*** **	**−.21*** **	**−.33*** **	1	**.36*** **	**.31*** **	**−.39*** **	**−.34*** **	**−.38*** **	−.08
(14) Health quality	**−.18*** **	−.06	.06	**.12** **	**.11** **	**.16*** **	.02	**.31*** **	−.03	**.15*** **	−.03	−.08	**.26*** **	1	**.44*** **	**−.25*** **	**−.32*** **	**−.23*** **	**−.11* **
(15) Sleep quality	**−.17*** **	**.11* **	.04	.06	.04	**.14*** **	.00	**.31*** **	.02	**.13*** **	−.07	−.08	**.25*** **	**.39*** **	1	**−.28*** **	**−.32*** **	**−.19*** **	**−.09* **
(16) Perceived stress	**.17*** **	**−.09* **	**−.25*** **	−.05	**−.23*** **	**−.32*** **	**−.11** **	**−.51*** **	−.02	**−.43*** **	**.32*** **	**.36*** **	**−.38*** **	**−.17*** **	**−.25*** **	1	**.45*** **	**.46*** **	**.11* **
(17) General anxiety	**.21*** **	**−.19*** **	**−.23*** **	−.03	**−.18*** **	**−.24*** **	**−.14*** **	**−.36*** **	.02	**−.31*** **	**.29*** **	**.38*** **	**−.28*** **	**−.16*** **	**−.23*** **	**.45*** **	1	**.43*** **	−.02
(18) Hopelessness	**.24*** **	**−.10* **	**−.27*** **	**−.09* **	**−.27*** **	**−.22*** **	**−.20*** **	**−.55*** **	.02	**−.42*** **	**.34*** **	**.33*** **	**−.37*** **	**−.18*** **	**−.14*** **	**.49*** **	**.40*** **	1	.05
(19) BMI	**.20*** **	.05	.01	**−.11** **	**−.12** **	−.03	-.05	**−.11** **	.03	−.06	.01	.03	**−.12** **	**−.10* **	−.07	.02	−.07	.03	1
M_{Men}	10.09	20.27	8.57	9.49	9.12	9.04	9.68	5.98	4.26	14.29	8.33	4.73	6.89	6.18	5.33	10.33	9.31	6.60	23.58
SD_{Men}	5.18	1.65	2.82	2.22	2.63	2.62	2.22	1.81	2.17	2.62	5.08	1.68	1.41	2.04	2.14	2.87	2.69	3.17	3.61
M_{Women}	9.54	20.39	9.16	9.91	9.60	7.98	10.14	5.75	3.40	13.79	10.16	4.77	7.07	6.01	5.14	11.21	10.11	6.61	21.36
SD_{Women}	4.82	1.71	2.95	2.28	2.69	2.79	2.22	1.80	2.02	2.68	5.59	1.73	1.36	2.11	2.17	3.00	3.03	3.26	2.98
T-test	1.84	−1.16	−3.46***	−3.14**	−3.04**	6.62***	−3.47***	2.15*	6.91***	3.21**	−5.81***	−0.30	−2.25*	1.38	1.50	−5.06***	−4.73***	−0.04	11.29***
Range	6–30	18–36	2–14	2–14	2–14	2–14	2–14	1–9	1–9	2–18	0–27	3–9	1–9	1–9	1–9	4–20	5–20	3–18	14.28–47.03

Note. [a] Measured with the six-item version of the mYFAS (after removing items 1–3). *p < .05; **p < .01; ***p < .001. *M* = mean; *SD* = standard deviation. Significant correlation coefficients are bolded.
